# Function of Circular RNAs in Fish and Their Potential Application as Biomarkers

**DOI:** 10.3390/ijms22137119

**Published:** 2021-07-01

**Authors:** Golam Rbbani, Artem Nedoluzhko, Jorge Galindo-Villegas, Jorge M. O. Fernandes

**Affiliations:** Genomics Division, Faculty of Biosciences and Aquaculture, Nord University, 8049 Bodø, Norway; golam.rbbani@nord.no (G.R.); artem.nedoluzhko@nord.no (A.N.); jorge.galindo-villegas@nord.no (J.G.-V.)

**Keywords:** aquaculture, biomarker, circRNA, growth, immunity, myogenesis, selective breeding

## Abstract

Circular RNAs (circRNAs) are an emerging class of regulatory RNAs with a covalently closed-loop structure formed during pre-mRNA splicing. Recent advances in high-throughput RNA sequencing and circRNA-specific computational tools have driven the development of novel approaches to their identification and functional characterization. CircRNAs are stable, developmentally regulated, and show tissue- and cell-type-specific expression across different taxonomic groups. They play a crucial role in regulating various biological processes at post-transcriptional and translational levels. However, the involvement of circRNAs in fish immunity has only recently been recognized. There is also broad evidence in mammals that the timely expression of circRNAs in muscle plays an essential role in growth regulation but our understanding of their expression and function in teleosts is still very limited. Here, we discuss the available knowledge about circRNAs and their role in growth and immunity in vertebrates from a comparative perspective, with emphasis on cultured teleost fish. We expect that the interest in teleost circRNAs will increase substantially soon, and we propose that they may be used as biomarkers for selective breeding of farmed fish, thus contributing to the sustainability of the aquaculture sector.

## 1. Introduction

Circular RNAs (circRNAs) are a class of long non-coding RNA molecules with a covalently closed-loop structure. They were first identified in the mid-1970s in plant viroids and hepatitis delta virus [1,2]. Until 2010, circRNAs were considered as byproducts of endogenous RNA splicing errors or the result of experimental flaws [3]. Recent advancements in biochemical protocols for RNA purification and computational methods enabled their *de novo* detection in RNA sequencing data across model and non-model organisms, thus propelling circRNA research [4,5,6].

**Figure 1:**
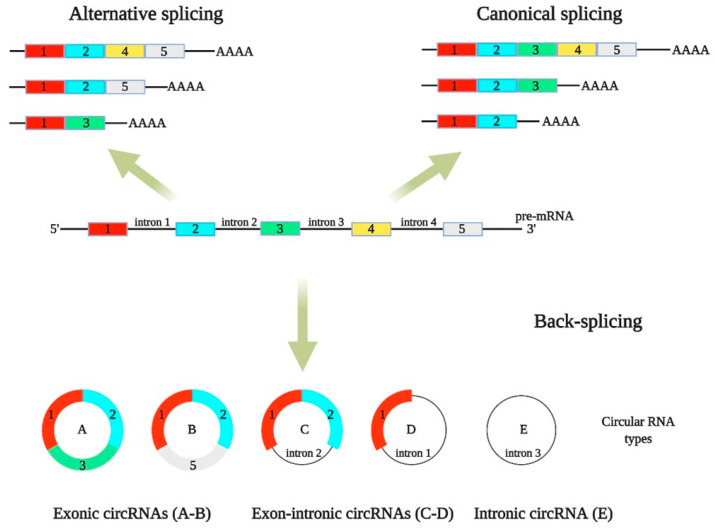
Biogenesis and diversity of circRNAs. CircRNAs are produced by a splicing process known as back-splicing. (**A**,**B**) Exonic circRNAs are derived from exons, (**C**,**D**) exon-intronic circRNAs are derived from both exons and introns, and (**E**) intronic circRNAs are derived from introns.

CircRNA biogenesis starts during pre-mRNA splicing, which is frequently coupled to transcription by RNA polymerase II [7,8]. They are generated by back-splicing, wherein the 3′ end of a downstream splice site ligates to the 5′ end of an upstream splice site. CircRNAs are divided in three categories: exonic, intronic, and exon-intronic circRNAs (Figure 1).

Exon-intronic circRNAs are formed by intron retention between exons during the back-splicing process. Many circRNAs contain multiple exons, which indicates that the back-splicing is largely connected with conventional splicing [9]. In most cases, cis-elements, trans-factors, and the spliceosome regulate circRNA back-splicing events [10,11,12]. CircRNAs can also be generated via a lariat-derived mechanism, involving a consensus motif with a 7-nt GU-rich element near the 5′ splice site and an 11-nt C-rich element close to the branchpoint site, which is located at a conserved distance from the 3’ splice site [13].

**Figure 2:**
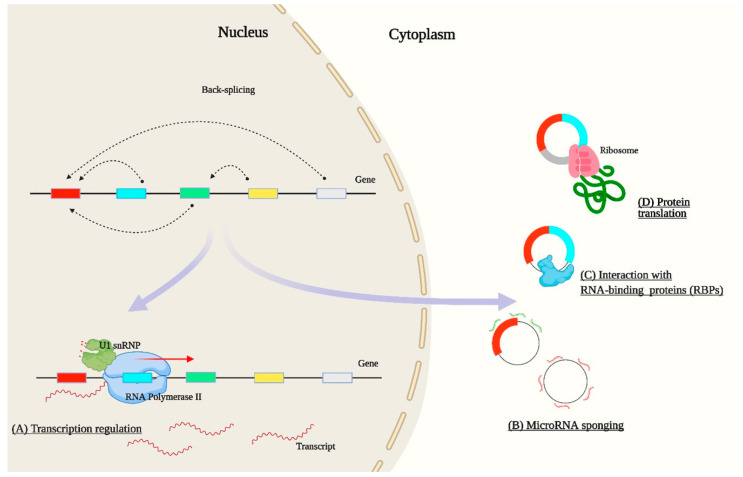
CircRNA functions. CircRNAs localized in the nucleus can regulate transcription (**A**), while their cytoplasmic counterparts can act as miRNA sponges (**B**), interact with proteins (**C**), and even regulate protein translation (**D**).

CircRNAs have unique properties. They lack free ends, which prevents their endonuclease- and exonuclease-mediated degradation in the cell and makes them resilient against numerous RNA turnover processes [14]. In different tissues, circRNAs have distinct temporal and spatial expression profiles, and many are evolutionarily conserved [14,15]. CircRNA conservation in mice and humans, obtained through tissue-specific enrichment, is 92.6%, 3.9%, 2.3%, 1%, and 0.1% in the brain, heart, liver, skin, and lung, respectively [16]. Their stability, expression pattern, and evolutionary conservation suggest that circRNAs have important regulatory roles in physiological and molecular processes (Figure 2). At the functional level, circRNAs have the capacity to regulate the expression of their linear counterparts by reducing the amount of pre-mRNA available for canonical splicing [5]. Furthermore, they can be co-expressed with their linear counterparts in the same locus [17]. Exon-intronic circRNAs (for example, circEIF3J and circPAIP2) can regulate transcriptional activity in the nucleus through interaction with RNA polymerase II and U1 small nuclear ribonucleoprotein at the promoter of their parental genes and enhance gene expression [7]. The function of circRNAs as miRNA sponges is the most common regulatory function studied across the species. They possess complementary sequences to bind to microRNAs, recognizing the seed regions and competitively deactivating them. CiRS-7 is among the best functionally characterized circRNAs that are highly expressed in the mammalian brain and it harbors more than 70 conserved matches to miR-7 [18].

A number of other circRNAs have been identified that contain a considerable number of target sites for miRNAs. Lately, it has been shown that mRNA is not the only target for miRNA, and many other non-coding RNAs also play an important role in miRNA-mediated gene expression regulation. CircRNA, lncRNA, mRNA, and pseudogenes can also have target sites for miRNAs. RNAs with the same miRNA binding site are able to compete for miRNA binding, thus functioning as competitive endogenous RNAs (ceRNAs). For example, Zhou et al. [19] described an hsa-circ-0034326/miR-25-3p/ITGA5 network that affects the development of hepatocellular carcinoma. It has also been reported that circRNAs have the potential to bind, store, and sequester RNA binding proteins to particular subcellular locations through sponges, decoys, scaffolds, and recruiters. Some classical examples of proteins containing circRNA binding sites are muscleblind, quaking, adenosine deaminases acting on RNA, nuclear factors NF90/NF110, and DExH-box helicase 9. CircMBL produced by the *muscleblind* gene contains multiple binding sites for the muscleblind protein and regulates its biogenesis [20]. Moreover, circRNAs may serve as mRNA to direct protein synthesis [21]. Yang et al. [22] have shown that circRNAs can contain many N-6-methyladenosine sites and a single site is sufficient to drive translation initiation.

Growing evidence suggests that the generation of a toxic circRNA from genomic translocation and dysregulation of a specific circRNA directly or indirectly influences the origin and development of cancer and other disorders [23]. The sensitivity and specificity of circRNAs makes them potential biomarkers for different human diseases [24,25,26,27]. CircHIPK3, circCDR1as, circPVT1, and hsa_circ_0013958 show differential expression in cancer tissues and have been considered as therapeutic agents for these cancers [28,29,30,31]. Long-term silica exposure increases circZC3H4 in alveolar macrophages and causes them to undergo proliferation and migration, which promotes the development of silicosis [32]. Furthermore, exosomal circRNAs have unique features reflecting the characteristics of tumors. Lower expression of CircSETD3 has been found to be correlated with larger tumor size in hepatocellular carcinoma tissue [33]. 

Over the past decade, the vast majority of circRNA studies have been conducted in human and model organisms, including zebrafish (*Danio rerio*) [34,35]. To date, there are only a handful of studies in non-model teleost fish, in spite of their importance from evolutionary and commercial perspectives. Ray-finned fish (Actinopterygii) represent more than 95% of all living fish species and make up roughly half of the extant vertebrate species. Teleostei (bony fish) is a major clade of Actinopterygii that constitutes the largest and most diverse group of vertebrates [36], which have acquired complex morphological and physiological features since they diverged from holosteans [37,38]. Several teleost species have been domesticated for food as well as for ornamental purposes. The common carp (*Cyprinus carpio*) is arguably the first fish species to have been domesticated, around 8000 years ago in Neolithic China, and it has become one of the leading farmed fish worldwide [39,40,41]. Nowadays, aquaculture plays a vital role in food security and economic stability, and it is in fact the fastest-growing farmed food sector worldwide as wild fish stocks become exhausted [42]. Carps, salmonids, cichlids, and tunas are the main fish groups extensively used in the aquaculture industry. Recent technological advances have enabled farmers to improve the biological efficiency of production through selective breeding. There are currently 104 breeding programs in aquaculture, most of them for teleost fish species [43]. It is estimated that the genetic gain for bodyweight varies from 2.3% to 42% per generation with an overall average of 12–13% [43,44,45]. Large-scale family-based breeding programs have been established in the industry for standard genetic improvement of aquaculture species, including Atlantic salmon (*Salmo salar*), rainbow trout (*Oncorhynchus mykiss*), Nile tilapia (*Oreochromis niloticus*), several carps (e.g., common carp (*Cyprinus carpio*), grass carp (*Ctenopharyngodon idella*), and silver carp (*Hypophthalmichthys molitrix*)), and Mediterranean species (e.g., gilthead seabream (*Sparus aurata*) and European sea bass (*Dicentrarchus labrax*)).

To date, none of these selective breeding programs consider circRNAs as biomarkers, despite their abundance and potential importance in teleost fish. By presenting the general knowledge of circRNAs in myogenesis and immunity achieved so far, this review presents in a digested manner our current knowledge of circRNAs in teleosts and anticipates their potential application in aquaculture research.

## 2. Bioinformatic Analysis of circRNAs

**Figure 3:**
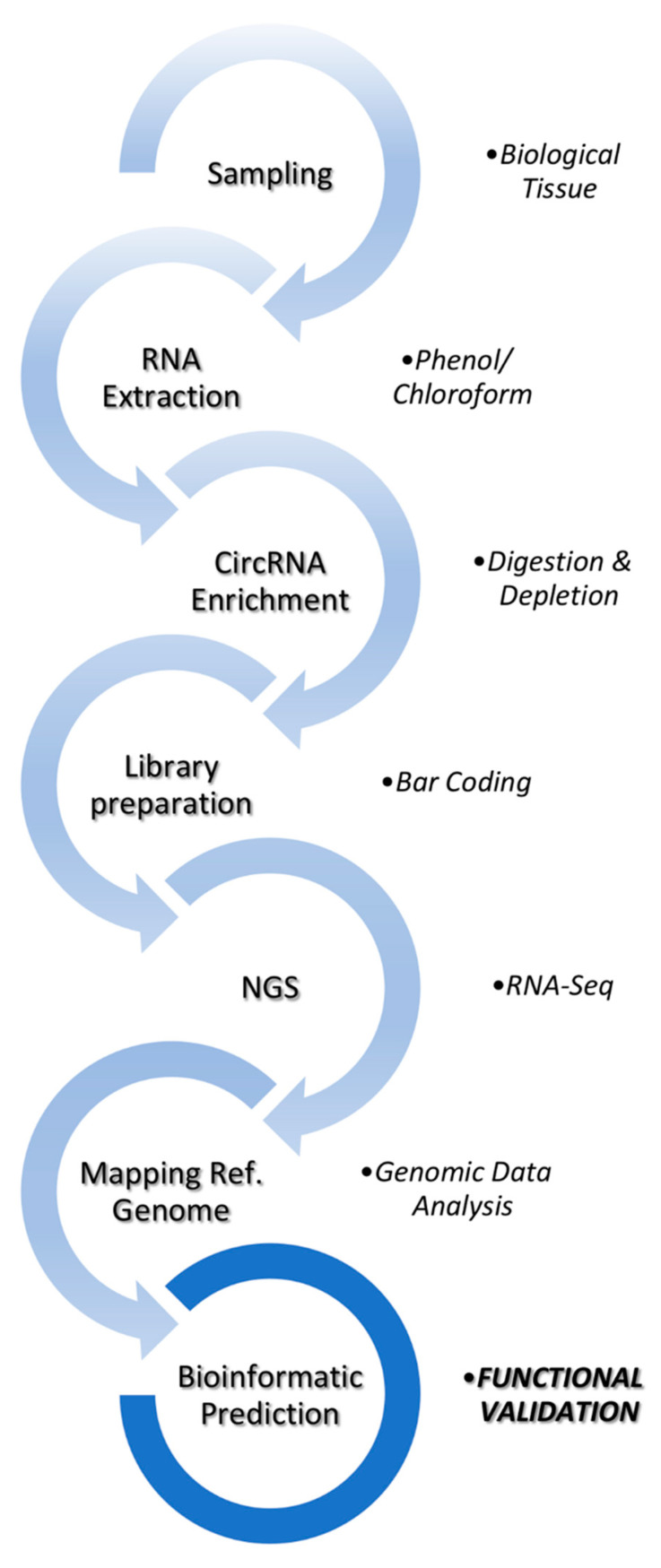
A workflow for circRNA library preparation and computational identification of circRNAs. Experimentally, this can be achieved from linear RNA and rRNA-depleted total RNA using high-throughput sequencing. Afterwards, circRNA can be identified with bioinformatic tools.

A schematic overview of the workflow for circRNA transcriptomics is represented in Figure 3. Total RNA must be digested with RNase R digestion or Rnase R and rRNA depletion before circRNA libraries are prepared. In order to identify circRNAs in model and non-model organisms, a variety of computational predictors have been developed. Some tools are specific to single-end or paired-end sequencing reads while others provide options to use either. CIRI2, find_circ, CIRCexplorer, KNIFE, CircCode, Uroborus, FUCHS, and DCC are among the mostly used tools in different organisms [10,21,46,47,48,49,50,51]. All these circRNA predictors are based on finding back-splicing junctions in RNA-seq data. However, they use different counting algorithms, and their output is extremely variable for the same transcriptome data. In addition, most of the algorithms detect circRNA without providing their full-length sequence. The degenerate sequence motifs at exon boundaries mean that a convolution of homology and sequencing errors can lead to false-positive alignments [52]. Some tools, such as ACFS and NCLscan, have been developed to overcome specific issues, including the presence of fusion and trans-splicing events [53,54]. Algorithm-specific false positives or low abundance circRNAs can be eliminated by combining the shared output from two or more algorithms. Comparing pairwise output from all possible combinations of algorithms, Hansen et al. [55] have shown that prediction reduces the fraction of RNase R-sensitive candidates from 10% to around 15%. However, predictors that use the same mapping tool are less suitable for comparisons that aim at reducing false positive rates. Zeng et al. [56] have reported that circRNA prediction tools are vulnerable to sequencing errors and strongly affected by sequencing depth.

To understand the function of circRNAs, it is important to know their host genes. CircParser can determine the host genes, the number of circRNA types from one host gene, and their minimum and maximum sizes in base pairs [57], while CircView can visualize circRNAs and quantify the number of samples expressing the predicted circRNAs [58]. Nowadays, deep learning is a rapidly developing area in the fields of machine learning and artificial intelligence. The primary advantage of using deep learning is that it allows combining several stages of information processing, such as feature selection and their classification [59]. Deep learning is expected to revolutionize biological fields such as genomics and epigenomics [60] and several simple deep learning models for circRNA prediction have been published in the last two years [61,62], but these methods still require additional experimental validation [63].

## 3. Role of circRNAs in Myogenesis and Growth

Muscle is a contractile tissue that constitutes approximately 30 to 80% of the body mass in vertebrates. It plays a crucial role in locomotion, metabolism, and homeostasis [64]. Muscle originates from the mesodermal germ layer of the embryo, which differentiates into skeletal, smooth, and cardiac muscle. The process by which animals form muscle tissue is known as myogenesis and it involves proliferation and differentiation of cells committed to the muscle lineage, known as myoblasts. Proliferation expands the myoblast cell population through cell division. After proliferation, daughter myogenic progenitor cells migrate through the muscle before leaving the cell cycle and entering terminal differentiation [65]. Differentiation requires withdrawal from the cell cycle and expression of key myogenic factors, leading to fusion of myogenic precursor cells into multinucleated myotubes [66].

During embryonic development, mesenchymal stem cells located in the mesodermal layer commit to the myogenic lineage and myoblasts fuse to form muscle fibers [67]. Later, at grow-out stages, a portion of myogenic cells become quiescent to form satellite cells and get activated following injury or growth stimulus [68]. Muscle mass changes rapidly due to an increase in myofiber size (hypertrophy) and number of myofibers (hyperplasia). In contrast to mammals, muscle development and growth in teleosts encompasses embryonic, larval, juvenile, and adult stages [69]. A variety of teleost species show two distinct modes of hyperplasia, namely stratified hyperplasia (restricted to the germinal zones only) and mosaic hyperplasia (dispersed throughout the musculature) [70]. These processes are regulated by a complex network of transcription factors, genes, signaling pathways, non-coding RNAs, and epigenetic modifications [71,72,73]. Among the key transcription factors involved in myogenesis are the myogenic regulatory factors (MRFs) myoblast determination protein, myogenic factor 5, myogenin, and myogenic factor 6, which have both specific and redundant functions [65].

Recent studies have reported circRNA expression in muscle across several vertebrate taxa, including teleosts, birds, and mammals (Table 1). The number of predicted circRNAs in both skeletal and cardiac muscle ranges from 622 to almost 38,000. The varied number of circRNAs in different species shows that they are abundant and dynamically expressed in muscle.

Muscle circRNAs are mostly derived from exons. A study on goat skeletal muscle tissue reported that 92.4% of circRNAs originated from exonic regions, which indicates their regulatory role at the post-transcriptional level [75]. Furthermore, functional clustering of circRNAs showed their association with different important signaling pathways, such as *Wnt/PI3K-Akt*, which is essential for muscle development [75]. In Nile tilapia only 622 distinct circRNAs were present in fast muscle. This number is comparatively lower than in other taxa but is likely due to a low sequencing coverage [57]. CircRNAs show temporal and spatial expression regulation during muscle development in mammals and the functional profiling of differentially expressed circRNAs has revealed their role in myoblast proliferation and differentiation processes. CircRNA290-miR27b-Fox3 and circRNA9210-miR-23a-MEF2C are two competing endogenous networks that have been found in skeletal muscles intricately linked to muscle fiber-type switching. However, the primary mechanism through which circRNAs are thought to regulate muscle growth in a murine muscle cell line [79] is their miRNA sponge function [83], since these circRNAs generally harbor one or more miRNA binding sites. To the best of our knowledge, there are no reports demonstrating the role of teleost circRNAs in myogenesis and elucidating the corresponding mechanisms involved.

A large number of circRNAs are expressed in muscle during ontogeny (Table 2). Circ-ZNF609 is a human circRNA derived from ZNF609 exon 2. Circ-ZNF609 is highly expressed in myoblasts and has been shown to promote myoblast proliferation [77]. It is also the first circRNA reported to be translated into a protein in a splicing-dependent and cap-independent manner, but the function of translated protein is still unknown. Additionally, the mouse orthologue circZfp609 inhibits myoblast differentiation by sponging out miR-194-5p [84]. *BCLAF1* is a novel target of miR-194-5p that affects circadian myoblast proliferation and myogenic fusion through stabilization of *Ccnd1* and *Tmem176b* mRNAs. In pigs, circTTN shows high expression both in embryonic and adult stages and regulates gene expression at the post-transcriptional level [82]. Luciferase screening and RNA immunoprecipitation revealed circTTN interaction with miR-432, leading to activation of *IGF2* expression. Binding of *IGF2* to its receptors induces autophosphorylation of tyrosine residues, which in turn activates the *PI3K/AKT* cascade that triggers myoblast proliferation [85]. Furthermore, circINSR identified in bovine muscle sequencing data is homologous to human circ-004869 [86]. It showed significantly higher expression in embryonic stages and its knockdown by siRNA reduced myocyte proliferation. CircINSR sponges out miR-34a, leading to expression of the proliferation gene *cyclin E2*. In contrast, functional characterization of circFUT10 revealed its role in myoblast inhibition and apoptosis [87]. Expression of circFUT10 inhibited myoblast proliferation by elevating the expression of serum response factor. Moreover, it also decreased expression of cell survival markers by binding to miR-133a, leading to myoblast apoptosis. The functions of some circRNAs in muscle development have also been investigated in chicken. In particular, circRBFOX2, circSVIL, and circFGFR2 have been reported to promote myoblast proliferation through interaction with different miRNAs. CircRBFOX2 originates from the *RBFOX2* gene, which can produce 11 different isoforms of circRBFOX2.2-4 [88]. Among them, circRBFOX2.2-3 and circRBFOX2.2-4 are differentially expressed in a temporal manner in embryonic muscle. A dual luciferase assay showed that inhibition of miR-206 by circRBFOX2 increases *ccnd2* expression, thus promoting myoblast proliferation. CircSVIL comprises exons 6 to 14 of *supervillin* and is expressed in early embryonic stages of muscle development [89]. A luciferase reporter assay and Ago2 RNA immunoprecipitation confirmed circSVIL and miR-203 interaction, which in turn increases *c-JUN* and leads to cell proliferation. Interestingly, circFGFR2 is involved in both myoblast proliferation and differentiation during early development of skeletal muscle in chickens [90]. RNAhybrid analysis revealed that circFGFR2 contained binding sites for both miR-133a-5p and miR-29b-1-5p, and their expression could prominently increase the numbers of cells that progressed to G0/G1 and reduced the numbers of S phase cells.

It is now apparent that several circRNAs expressed in myoblasts regulate the cell cycle and muscle differentiation through several mechanisms in higher vertebrates. In Qinchuan cattle, circSNX29 functions as a ceRNA and its overexpression sequesters miR-744 away from *Wnt5a* to increase the expression of Wnt5a and phosphorylation of *PKC*, thereby activating the *Wnt5a/Ca2+/CaMK-IIδ* pathway and inducing myoblast differentiation [93]. On the other hand, circHUWE1 modulates the protein level of phosphorylated (p-)Akt (S473), resulting in inhibition of myogenic differentiation. A dual-luciferase reporter assay and AGO2 RNA immunoprecipitation showed that circHUWE1 can sponge out miR-29b, leading to myoblast proliferation through AKT activation [94]. It has also been reported that overexpression of circLMO7 inhibits myoblast differentiation and apoptosis by functioning as a competing endogenous RNA for miR-378a-3p [74]. CircLMO7 effects the cell cycle by increasing the number of myoblasts in the S-phase of the cell cycle and decreasing the proportion of cells in the G0/G1 phase. In chickens, miR-30a-3p and circHIPK3, which are both produced by the third exon of the *hipk3* gene, may have opposite effects on myoblast differentiation [95]. miR-30a-3p can inhibit the differentiation of myoblasts by binding to *myocyte enhancer factor 2C*, but circHIPK3 contains three potential binding sites for miR-30a-3p and could therefore counteract its effect. 

To date, the role of circRNAs in muscle development and growth in teleost is poorly understood. In fact, the few available reports only profiled circRNAs expressed in muscle without any functional assays. In zebrafish, 376 and 523 circRNAs were found to be expressed in skeletal muscle and heart, respectively [34]. The circRNAs expressed at highest levels in skeletal muscle and heart were circ_ttnb1 and circ_furina, respectively. Nedoluzhko et al. [57] have reported that many host genes of circRNAs predicted in Nile tilapia were related to myogenesis and muscle function, including *calcium/calmodulin-dependent protein kinase*, *troponin T3* and *myocyte-specific enhancer factor 2C*. This limited evidence suggests their involvement in teleost muscle growth, but functional studies are needed to validate this assumption. This fundamental knowledge will provide valuable insights into novel biomarker approaches to increase aquaculture production and sustainability, as further discussed in this review.

## 4. CircRNAs as Regulators of the Immune System

A vast diversity of sophisticated defense mechanisms that can distinguish self from non-self characterize the immune system of all multicellular organisms [96]. Immunity has been historically divided into innate and adaptive branches, with vertebrates possessing both and invertebrates having only innate immune cells, which express, as a hallmark, classical conserved receptors that bind molecular patterns contained within a variety of signaling elements [97].

New studies in mammals have suggested that circRNAs in macrophages can lead to macrophage activation, resulting in polarization and the induction of potent innate immune responses mediated by inflammation. Following a microarray analysis of bone marrow-derived macrophages polarized with classical Th signature molecules (*IFN- γ* and *IL-4*), 189 differentially expressed circRNAs were recorded [98]. CircRNA-010231, circRNA-003780, and circRNA-010056 were highly expressed in M1 cells, while circRNA-001489, circRNA-013630, circRNA-003424, and csircRNA-018127 were more abundant in the remaining M2 fraction. Bioinformatic tools were used to uncover the potential link of the obtained putative targets with specific miRNAs predicting. The results indicated that miR-1964-5p, miR-141-5p, miR-19b-2-5p, miR-6950-5p, and miR-145a-5p have potential roles in activating macrophages towards a pro-inflammatory M1 phenotype.

It is widely accepted that circRNAs actively participate in eliciting antiviral immune responses, but their specific mechanisms are still under debate. Efforts to understand the governing mechanisms unmasked the presence of the RNA modification N6-methyladenosine (m6A), which acts as a “self” marker and abrogates circRNA immunity [99]. Moreover, the same authors provide evidence that unmodified circRNAs and K63-polyubiquitin activate *RIG-1* and innate immune signaling. Besides, tethering of the m6A reader protein YTHDF2 to unmodified circFOREIGN is sufficient to avoid circRNA immunity. Conversely, Wesselhoeft et al. [100] put forward a contradictory view, as these research teams suggest that unmodified exogenous circRNAs are able to bypass cellular RNA sensors and thus do not induce any immune response, mediated or not, by *RIG-1* and TLR in vitro and in vivo. In the meanwhile, we can comment on parallel mechanisms dealing with RNA back-splicing at the cell nucleus that modulate the viral resistance of the resulting circRNA. The positive regulation is achieved by the nuclear factor 90 and splice variant NF110 (NF90/NF110) [101], while the helicase DHX9 and the RNA-editing enzyme ADAR induce a negative regulation acting as destabilizers between intronic repeat sequences [102]. Conversely, upon viral infection, RNase L, a widely expressed cytoplasmic endoribonuclease, is activated via an unknown mechanism and degrades these circRNAs, thus activating the dsRNA-dependent kinase (*PKR*) for an innate immune response [103,104]. Finally, in addition to *PKR*, recent work suggests that a circRNA may similarly inhibit the activity of cyclic GMP-AMP synthase (cGAS), a cytosolic DNA sensor [105].

Molecular and physiological evidence indicate that circRNAs are abundantly expressed and perhaps functionally active in patients presenting multifactorial polygenic chronic diseases, such as metabolic autoinflammatory type 2 diabetes [106], acute pancreatitis [107], systemic lupus erythematosus [108], malignant hepatocellular carcinoma [109], and gastric cancer [110]. The close relationship between circRNAs and critical blood cells involved in host defense affects their intrinsic defensive properties following series of pivotal events under diverse pathological conditions, and some circRNAs (e.g., circFUNDC1, circPDS5B, and circCDC14A) have recently been proposed as biomarkers for diagnosis and prognosis of acute ischemic stroke and inflammation [111]. Despite the complexity of these molecular pathways, we feel that the next years will unveil many more exciting findings due to the extended new sequencing techniques and sophisticated bioinformatics tools developed every day in various laboratories around the world.

Increasing evidence suggests that a complex RNA network is involved in the teleost immune response (Table 3). CircPIKfyve, a novel circRNA derived from PIKfyveact, plays a crucial role in innate immune response in teleost fish [112]. CircPIKfyve formation is regulated by RNA binding protein QKI. It functions as a ceRNA to absorb miR-21-3p, thereby increasing the abundance of mitochondrial antiviral signaling protein and activating the downstream *NF-κB/IRF3* pathway to enhance the antiviral response.

The role of circRNAs in the antiviral response has been comprehensively studied in carp species and a mRNA–miRNA–circRNA network has been identified in hemorrhagic disease caused by grass carp reovirus (GCRV). Grass carp hemorrhagic disease (GCHD) is one of the most serious diseases in the freshwater aquaculture industry because of the lack of a commercially available vaccine. He et al. [113] reported 72 binding miRNAs for 41 differentially expressed circRNAs. Among the targeted genes, 8 were found to be involved in blood coagulation, hemostasis, complement activation and coagulation downstream signaling; their upregulation might have led to endothelial and blood cell damage and hemorrhagic symptoms. Based on the miRNA binding network, it was proposed that circRNA-2780/cik-miRNA-7796/KAT6B, circRNA-1351/cik-miRNA-10648/B2L14, and circRNA-1591/cik-miR-8141/AMPD3 interactions are likely associated with GCRV infection [114]. Pan et al. [115] investigated the expression of circRNAs in this GCRV virus, and their potential role in viral infection. Viral dsRNA can generate vcircRNAs (GCRV-derived circRNA) via unknown splicing machinery in the host that limits virus infection and sponges host or virus miRNAs. By integrating data of 32 vcircRNAs, 1685 host miRNAs, and 786 host mRNAs, the authors showed that four vcircRNAs sponge four host miRNAs that target 26 host mRNAs. Additionally, vcircRNAs into kidney cells suppressed the expression levels of the *ns4* gene and the *VP7 outer capsid glycoprotein.*

The circRNA landscape following bacterial infection in Japanese flounder (*Paralichthys olivaceus*) and Nile tilapia has been investigated to understand the involvement of circRNAs in the antibacterial immune response [116,117]. *Edwardsiella tarda* infection affects a network of multiple combinations of circRNAs, miRNAs, and mRNAs in Japanese flounder. This complex network contained 198 circRNA–miRNA pairs and 3873 miRNA–mRNA pairs, including 44 circRNAs, 32 miRNAs, and 1774 mRNAs. In Nile tilapia, the expression profiles of circRNAs revealed dynamic changes associated with the course of *Streptococcus agalactiae* infection. The presence of mir-221 and mir-222 in connection with 10 and 11 circRNAs, respectively, suggests a link between circRNAs and miRNAs that facilitates the immune response through the expression of immunomodulatory proteins in the brain of *S. agalactiae* infected Nile tilapia. The authors coined the term neurimcircRs to denote circRNAs that modulate both neuronal and immune processes.

## 5. Advancements in Teleost circRNA Research

Teleost circRNAs have their own distinctive features compared to higher vertebrates. In particular, the GT/AG or GU/AG canonical splicing mechanism has been prominently observed in some teleost species. The splicing sites of nearly all circRNAs identified in grass carp contain GT/AG [114], whereas in crucian carp, circRNAs are characterized by a GU–AG splicing site [118]. Furthermore, several zebrafish circRNAs have been identified as orthologues of human circRNAs, which suggests conservation of their biological function and regulatory role in teleost fish [34]. Lui et al. [119] have reported that the 1028 circRNAs expressed throughout early development are involved in circRNA–miRNA–mRNA networks that may modulate embryonic differentiation in zebrafish. The genome-wide identification of 3868 circRNAs revealed their widespread distribution in previously well-annotated protein-coding and long non-coding RNA gene loci [34,35], and it is becoming apparent that they are ubiquitously expressed in various teleost tissues. Furthermore, a comparison between blood, brain, heart, gills, and muscle circRNAs revealed that 78.6% have tissue-restricted expression.

Among aquaculture species, one of the earliest studies in teleosts providing a transcriptome-wide identification of circRNAs was performed in large yellow croakers (*Larimichthys crocea*) [120]. The RNA-seq datasets of 72 individuals were analyzed for the presence of circRNAs and further experimentally validated. Predictions revealed 975 putative circRNAs, of which 65% were from exonic regions. The authors also reported 151 alternative splicing circularization events in putative exonic circRNAs from 67 unique parental gene loci. Functional annotation showed that the genes hosting circRNAs were mainly classified into cell (6.8%), metabolic process (5.3%) and catalytic activity (3.4%). Significantly enriched KEGG processes were related to small molecule binding, ATP binding, the macromolecule metabolic process, the cellular metabolic process, translation factor activity, and translation initiation factor activity. Regulation of energy metabolism is closely related to cell cycle progression. It has been suggested that regulation of metabolic pathways in response to growth-factor-initiated signaling facilitates cell growth and proliferation [121]. These results are particularly relevant in establishing promising new avenues of research in teleost circRNAs and muscle growth.

Cyprinidae are the most studied teleost family regarding the presence, distribution, and putative function of circRNAs. The 5052 circRNAs identified in grass carp ranged in length from 150 to 59,886 bp but most were smaller than 2000 bp [113]. Liu et al. [114] reported 76 circRNAs, 798 mRNAs, and 186 miRNAs differentially expressed with GCRV infection. In crucian carp (*Carassius auratus gibelio*), 37 circRNAs were found in the kidney. They are thought to have important biological functions related to catalytic activity and binding. KEGG analysis of these circRNA host genes revealed their link to Wnt and MAPK signaling pathways, as well as to ubiquitin-mediated proteolysis [118].

In Japanese flounder, 5478 circRNAs have been reported and their average length was higher than in other teleost fish species, since 78% were as large as 5000 bp. In total, 62 circRNAs were differentially regulated with *E. tarda* infection [117]. By comparing brain samples from healthy and S. agalactiae-induced meningitis in Nile tilapia, Fan et al. [116] identified a total of 11,263 novel circRNAs and 76% had an exonic origin. Differentially expressed circRNAs, such as Oni_circRNA_002423, Oni_circRNA_002834, Oni_circRNA_006192, Oni_circRNA_007265, Oni_circRNA_000305, and Oni_circRNA_006456, are thought to target miR-221, miR-222, miR-183, and miR-124, thus suppressing their expression in response to meningoencephalitis in Nile tilapia. It is particularly interesting to note that 14 circRNAs in Nile tilapia have similar sequences to those present in coelacanth (*Latimeria chalumnae*), indicating circRNA sequence (and perhaps function) conservation among phylogenetically distant ray- and lobe-finned fishes.

Recently, a heat stress-related circRNA network has been described in rainbow trout. Due to the increase in ocean temperature, rainbow trout suffer from varying degrees of thermal stress, which leads to mass mortality and severely restricts their production. Quan et al. [122] have reported novel_circ_003889-novelm0674-3p-hsp90ab1, novel_circ_002325-miR-18-y-HSPA13, and novel_circ_002446-novel-m0556-3p-hsp70 ceRNA networks involved in development of the molecular response to relieve heat stress. The significant differences in expression levels of HSP70 and HSP90 families under heat stress suggest that these protein families are involved in important regulatory pathways in the organismal response to heat stress.

Despite some notable recent advancements, the functional role of circRNAs in teleosts is still poorly understood, especially in relation to muscle growth. We expect that the exciting evolutionary history of teleost fish, as well as their commercial importance, will stimulate circRNA research in the near future.

## 6. Potential Application of circRNAs in Aquaculture

The application of circRNAs in aquaculture is still in its infancy, mainly due to the lack of fundamental knowledge in this field. Nowadays, the circRNA sponge function has caught significant interest because the targeted dysregulation of miRNA expression is a promising strategy for the development of novel types of therapies for human conditions, as well as high-value biotechnological products. The latter include improved breeds of agricultural plants and animals and even farmed fish and shellfish species.

One of the main traits of interest for the global aquaculture industry is fast and enhanced fish muscle growth, which is currently targeted mainly through diet improvements and selective breeding [123,124]. As discussed above, several miRNAs and signaling pathways can influence growth and muscle development in fish. In particular, miR-1, miR-133a-3p, and miR-206 are upregulated in adult common carp and Nile tilapia compared to juveniles [125,126]. These miRNAs reduce the expression of growth-promoting genes, thus reducing growth rate at the adult stage. Some other miRNAs function as repressors of negative regulators of muscle development, such as miR-181b-5p which regulates *myostatin b* expression in genetically improved farmed tilapia [127]. The application of miRNAs and other linear RNAs for prolonged dysregulation of molecular pathways in order to promote muscle growth has significant disadvantages related to the short lifetime of these linear single-stranded molecules in cells and tissues. To this aim, it seems preferable to use circRNAs as modulators of muscle growth through targeted miRNA silencing, since they are more stable than linear RNAs [128].

**Figure 4:**
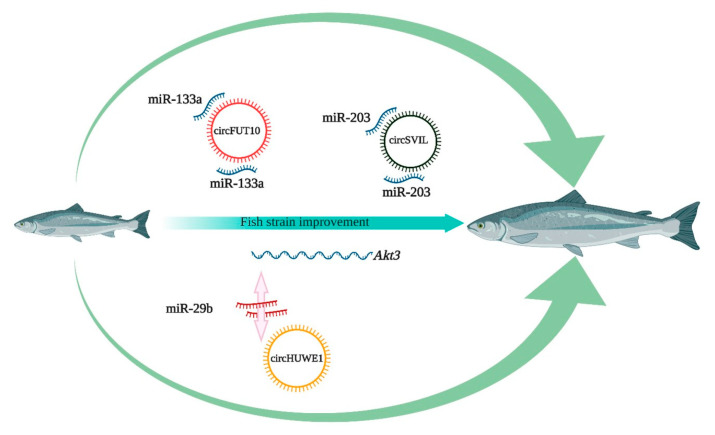
Proposed application of circRNAs in improved fish growth using their miRNA sponge and ceRNA properties.

A number of miRNA gene families are evolutionarily conserved between higher vertebrates and teleosts. More importantly, their targets show conservation among related species, suggesting that the same conserved miRNAs can be expected to regulate pathways and biological processes among evolutionarily divergent species. Many interconnected signaling pathways (e.g., Wnt and PI3K-AKT) are evolutionarily conserved and orchestrate muscle development in many species. After mining the Nile tilapia miRNA database, we identified three miRNAs (miR-203, miR-133a, miR-29b) that are already known in mammals as the targets of circSVIL, circFUT10, and CircHUWE1, respectively. Moreover, these circRNA host genes are also reported in other teleost species. Hence, we propose that these circRNAs are promising candidates to improve muscle growth in farmed finfish (Figure 4). Some other circRNAs of interest are significantly expressed in grass carp, crucian carp, and Nile tilapia: cid_circ_2040, novel_circ_0003296, circRNA-1351, and Oni_circRNA_002834 [115,118,119]. Cid_circ_2040 has been shown to bind miR-196b and miR-216a, which are well known for their role in muscle regulation. In Nile tilapia, Oni_circRNA_002834 binds to miR-221, miR-222, and miR-734 and was significantly upregulated in brain with meningitis. The above-mentioned circRNAs associated with immune and muscle regulatory networks are relevant subjects for future research to improve fish fitness and growth.

A quantitative trait locus (QTL) is a genome region that is associated with a particular phenotypic trait that is determined by several genes. This information can be explored to select for favorable alleles and, to date, quantitative trait loci (QTLs) for many important traits have been mapped in farmed fish, including cold and salinity tolerance, sex determination, growth traits, and disease resistance [129,130]. Liu et al. [131] have categorized 10, 7, and 8 significant QTLs for body weight, total length, and standard length in Nile tilapia. In farmed land animals, circRNAs are known to be significantly enriched in QTLs related to traits of commercial importance, such as growth and flesh quality [132]. For example, circRNA227 has been localized in chromosome 6 within the interval of QTLs for intramuscular fat content and loin muscle area in pigs. Virtually nothing is known about polymorphisms (e.g., single nucleotide polymorphisms (SNPs)) of circRNAs at the structural or functional level in fish. Nevertheless, it has been demonstrated in humans that circRNA expression is affected by local SNPs, which are referred to as circQTLs [133]. Structurally, these circQTL SNPs are present in both the back-splicing site and circRNA regions, and they have been linked to genome-wide association study signals of complex diseases [134].

CircQTLs provide a new scope in selective breeding schemes to improve the accuracy of selection for more robust aquaculture species. Moreover, the successful targeting of non-coding sequences using the CRISPR/Cas9 system in commercially important fish [135] has paved the way for genome editing of key circRNAs that may result in improved phenotypes for traits of interest, such as growth and disease resistance.

## 7. Major Challenges of circRNA Research in Aquaculture

The discovery of back-splicing junctions depends on the detection of potential splice sites through mapping sequencing reads back to the reference genome [21]. Thus, detection performance of the algorithms is highly dependent on the splice alignment against a reliable genome. In teleosts, only a few species have a high-quality genome assembly, which could be a bottleneck in the discovery of true circRNAs. In addition, genome annotation is another constraint, as there is an even lower number of species with a sound genome annotation. Using de novo prediction tools without prior knowledge of exon-intronic annotations could increase the rate of false positive circRNAs, as novel and unannotated splice sites are likely to be spurious [55]. Thus, additional validation is required to confirm circRNAs in teleosts. Species differences in splicing detection is another challenge among aquaculture species and, to date, there is no database of teleost circRNAs.

As mentioned above, the performance of circRNA detection tools negatively correlates with sequencing error [56]. Since there is no benchmark protocol to prepare circRNA libraries, variation in RNA library preparation methods (e.g., size selection) significantly affects the abundance of circRNAs in the resulting RNA-seq datasets. Biochemical reagents for library preparation are mainly designed for mammals, which could also limit the potential output of circRNAs in teleosts. Thus, there is a need for standardization and caution should be taken during circRNA library preparation.

## 8. Conclusions

CircRNAs show some degree of conservation across taxa and are ubiquitously present in teleost fish. They modulate gene expression through several mechanisms (e.g., miRNA sponging) and their expression has been linked to immunity and myogenesis. Understanding the role of teleost circRNAs in these biological processes will enable their application as growth and health biomarkers in aquaculture. However, many challenging questions remain to be answered. We expect that future studies will address key gaps of knowledge, namely the efficiency of in vivo RNA circularization and the precise function and involvement of circRNAs in molecular pathways. Only then will we be able to develop novel circRNA-based biotechnology tools (e.g., genome editing) to promote new directions for selective breeding of commercially important species and sustainable aquaculture production.

## Figures and Tables

**Table 1:** Overview of circular RNA expression in vertebrate muscle. The type of RNA treatment used during the library preparation is indicated, along with the number of circRNAs detected in silico and experimentally validated by qPCR.

Species	Common Name	Tissue/Cell Type	RNA Treatment	Detected circRNAs	Validated circRNAs	Reference
*Bos taurus*	Cattle	Skeletal muscle	RNase R+ rRNA−	12,981	17	[74]
*Capra aegagrus hircus*	Wild goat	Skeletal muscle	RNase R+ rRNA−	9090	4	[75]
*Gallus gallus*	Chicken	Skeletal muscle	RNase R+ rRNA−	13,377	8	[76]
*Homo sapiens*	Human	C2C12 myoblasts	rRNA−	2175	31	[77]
*Macaca mulatta*	Rhesus monkey	Primary myoblasts	RNase R	2100	29	[78]
*Mus musculus*	House mouse	C2C12 myoblasts	RNase R+ rRNA−	37,751	10	[79]
		C2C12 myoblasts	rRNA−	1592	31	[77]
*Oreochromis niloticus*	Nile tilapia	Skeletal muscle	rRNA−	622	-	[57]
*Ovis aries*	Sheep	Skeletal muscle	RNase R+	6000	10	[80]
*Sus scrofa*	Pig	Skeletal muscle	rRNA−	4402	2	[81]
		Skeletal muscle	RNase R+ rRNA−	7968	6	[82]

**Table 2:** A summary of circRNAs involved in skeletal muscle growth, showing their name, species, and tissue of origin, along with their biological roles and modes of action.

CircRNA	Species	Tissue/Cell	Biological Role	Mode of Action	Reference
Circ-ZNF609	*Homo sapiens*	C2C12	Myoblast proliferation	Protein encoding	[77]
CircTTN	*Bos taurus*	Skeletal muscle		miRNA sponge	[91]
CircINSR	*Bos taurus*	Skeletal muscle		miRNA sponge	[86]
CircFUT10	*Bos taurus*	Skeletal muscle		miRNA sponge	[87]
CircSVIL	*Gallus gallus*	Skeletal muscle		miRNA sponge	[89]
CircFGFR2	*Gallus gallus*	DF-1		miRNA sponge	[92]
CircRBFOX2	*Gallus gallus*	Skeletal muscle		miRNA sponge	[88]
CircSNX29	*Bos taurus*	Skeletal muscle	Myoblast differentiation	miRNA sponge	[93]
CircHUWE1	*Bos taurus*	Skeletal muscle			[94]
CircLMO7	*Bos taurus*	Skeletal muscle			[74]
CircHIPK3	*Gallus gallus*	Skeletal muscle			[95]

**Table 3:** CircRNAs associated with teleost immunity and differentially expressed after viral or bacterial infection.

Species	Common Name	Infection Type	Tissue	DE * circRNAs	Reference
*Ctenopharyngodon idellus*	Grass carp	Viral	Spleen	41	[113]
			Kidney	76	[114]
			Kidney	-	[115]
*Oreochromis niloticus*	Nile tilapia	Bacterial	Brain	837	[116]
*Paralichthys olivaceus*	Japanese flounder	Bacterial	Intestine	62	[117]

* DE: differentially expressed.
